# Near and long-term perspectives on strategies to decarbonize China’s heavy-duty trucks through 2050

**DOI:** 10.1038/s41598-021-99715-w

**Published:** 2021-10-14

**Authors:** Nina Khanna, Hongyou Lu, David Fridley, Nan Zhou

**Affiliations:** International Energy Analysis Department, Lawrence Berkeley National Laboratory, One Cyclotron Road MS90R2121, Berkeley, CA 94720 USA

**Keywords:** Climate-change mitigation, Climate-change policy, Energy and society

## Abstract

China needs to drastically reduce carbon dioxide (CO_2_) emissions from heavy-duty trucks (HDTs), a key emitter in the growing transport sector, in order to address energy security concerns and meet its climate targets. We address existing research gaps by modeling feasibility, applicability, and energy and emissions impacts of multiple decarbonization strategies at different points in time. China still relies heavily on coal power, so impacts of new HDT technologies depend on the timing of their introduction relative to progress toward non-fossil power. We use a bottom-up model to simulate HDT energy consumption and CO_2_ emissions through 2050. Results show that beginning to deploy battery electric and fuel-cell HDTs as early as 2020 and 2035, respectively, could achieve significant and the largest CO_2_ emissions reduction by 2050 with a decarbonized power sector. However, viable near-term strategies—improving efficiency and logistics, switching to liquefied natural gas—could halve HDTs’ current diesel consumption and CO_2_ emissions by 2050. Our results underscore the need for a mix of near- and long-term policy and technology options to decarbonize China’s HDTs.

## Introduction

Heavy-duty trucks (HDTs) in the road freight sector are a growing area of focus for reducing transportation-related oil consumption and greenhouse gas emissions because of this sector’s disproportionate environmental impacts and the technical challenges of mitigating them. HDTs account for approximately 10% of all vehicles but 70% of road freight, 50% of all trucking energy consumption, and 40% of total transportation-related carbon dioxide (CO_2_) emissions^1,2^. Existing options for reducing HDTs’ CO_2_ impacts include energy-efficiency improvements and switching to cleaner fuels such as liquefied natural gas (LNG) and cleaner low- or zero-emission technologies such as battery electric, catenary or in-road dynamically charging electric, and hydrogen fuel cells.

Globally, established multi-national manufacturers and start-ups have been developing electric HDT prototypes since 2016, with 10 battery electric truck models slated for commercial deployment by 2021^3,4^. Although electric vehicle battery costs have declined significantly in recent years, HDTs’ performance and operating characteristics place significant demand on battery capacity, durability, and number of discharge cycles^1^. Current HDT battery costs range from US$175 to $375/kilowatt-hour (kWh) but could drop to less than US$100/kWh by the 2030s^1,5,6^. Some manufacturers are developing prototypes for fuel-cell trucks, with pilot production anticipated in 2022, and three European companies are working on dynamically charging trucks using catenary lines, on-road rails, and induction. Fuel-cell trucks use hydrogen produced from natural gas by methane reforming or by electrolysis of pure water. The cost of a fuel-cell truck is anticipated to decrease from the current level of US$256,000–$480,000 to US$150,000–$200,000 by 2030^1,2,6^.

China is the world’s largest HDT market, dominated by trucks used for specialized applications in urban areas and by long-distance tractor-trailers for freight transport. The vast majority of China’s HDTs are diesel-powered. LNG and battery-electric vehicles have recently started to increase in number in urban settings but still represent less than 2% of total stock^7^. China’s road freight sector is decentralized, with 70% of vehicles belonging to private, individual owners and an average of only 3.2 vehicles per carrier^8^. Combined with older diesel vehicles that are also underpowered and heavier, China has recognized the need to improve overall HDT efficiency with adoption of fuel economy standards for selected categories of HDTs in 2011 and subsequent revisions. Since 2015, China’s central government has been promoting battery electric and fuel-cell vehicles through broad subsidies and favorable transport policies for these “New Energy Vehicles (NEVs)”. In April 2020, NEV subsidies for HDTs were extended through 2022 with only small (10% and 20%) annual reductions in subsidy amounts^9^. In light of China’s national and international energy and climate mitigation targets, including its most recently announced goal to peak its total CO_2_ emissions before 2030 and to achieve carbon neutrality by 2060, NEVs are expected to play a critical role in supporting national plans to phase out all internal combustion engines, likely by 2050, meet its short-term oil caps and CO_2_ peaking goal and accelerate a peak and decline in the country’s oil consumption. In the near term, NEV HDTs are also being targeted by stimulus policies to support transport electrification as part of China’s post-Covid-19 economic recovery.

### Multiple near-term strategies exist for decarbonizing HDTs in China

In the near term, there is significant potential to increase the overall energy efficiency of China’s HDTs through improvements in engine efficiency, lightweighting, and aerodynamic improvements based on current market conditions. Because Chinese truck engines are still underpowered and smaller than those used in HDTs in the U.S. and EU markets, engine performance and efficiency could improve with adoption of technologies such as advanced turbochargers, combustion system optimization, turbo-compounding, advanced engine control, on-demand accessories, after treatment improvements, engine friction reduction, and high-efficiency selective catalytic reduction systems^10^.

China’s HDTs are 10% heavier than comparable vehicles in other markets; in particular, key components of vehicle frames and suspension springs are 30–40% heavier than those of comparable vehicles^11^. Only 20% of China’s tractor-trailers have a traction ratio (a measure that indicates fuel efficiency, with a higher ratio representing better fuel efficiency) greater than 5.0 while 50% of China’s tractor-trailers have a traction ratio of 4.5 to 5.0^12^. Forty-three percent of straight trucks in China have a payload ratio (a measure of fuel efficiency where a higher value indicates better efficiency) greater than 1.6, and more than 25% of straight trucks have a ratio below 1.0^12^. Lightweighting technologies and materials such as high-strength steel and aluminum could help reduce vehicle weight while increasing fuel efficiency.

Technologies to improve aerodynamics can also increase fuel efficiency. Trailer side fairings or “skirts,” which are already commonly used in the U.S. and Canada, are just beginning to be adopted in China. Low rolling resistance tires can also save significant fuel, but adoption in China is still low^13^.

LNG-fueled trucks are an alternative to conventional diesel HDTs, providing comparable driving range between refueling with reduced CO_2_ and air pollutant emissions. Compared to diesel fuel, approximately double the volume of LNG is needed to travel the same distance^1^ and to provide comparable horsepower, acceleration, and cruise speed^14^. The number of LNG refueling sites in China tripled from 610 sites in 2012 to 2552 in 2018^15^. The time required to refuel LNG trucks is similar to the time needed to refuel diesel trucks, but LNG refueling stations are more complex and require specialized equipment and driver training.

The technology and cost-effectiveness of LNG engines for HDTs have been continually improving, with the marginal cost of LNG HDTs declining compared to the cost of diesel HDTs. The difference in up-front purchase costs for LNG and diesel HDTs declined from 120,000 to a range of 80,000–100,000 renminbi (RMB) (or 60,000–80,000 RMB if compliance with National VI emission standards is considered) by 2016^15^. Industry experts expect the LNG purchase price difference to be further reduced to only 30,000 RMB in 2020^15^. From a life-cycle-cost-of-ownership perspective, LNG trucks are more cost-competitive than diesel trucks because of significant fuel-cost savings that more than offset the incremental capital cost. By 2020, the total life-cycle-cost-of-ownership over 10 years for LNG trucks is projected to reach US$500,000, compared to US$700,000 for diesel trucks^2^. However, if the cost of additional necessary infrastructure such as refueling stations is considered, then LNG trucks will cost more than diesel trucks^1^.

Multiple systemic improvements in freight operations and logistics can help reduce fuel use and emissions growth, but most of these improvements have not been realized because of existing technical, economic, and/or political barriers and lack of enabling mechanisms or policies to overcome these barriers. Globally, logistics measures have been implemented at various geographical scales including by municipalities, subnational regions, national or regional trading blocs, and by freight operators when benefits outweigh costs^1^. In China where over- and under-loading are currently major challenges, back-hauling by delivering cargo on return trips is a key strategy for improving vehicle utilization and decreasing vehicle travel activity. Optimized routing and co-loading can improve operational efficiency of HDT fleets, and driver training and feedback and platooning can further reduce fuel consumption.

### Adoption of battery electric and hydrogen fuel-cell heavy-duty trucks depends strongly on costs and technological commercialization

The physical characteristics and operation of long-haul HDTs make them more difficult to electrify compared to light-duty vehicles. Technical constraints include heavier weight and larger vehicle size, the need to withstand more rugged operation, and longer travel distances and operating times. Compared to the requirements for light-duty vehicles, these defining characteristics of HDTs require batteries with greater energy densities, higher specific power, greater durability, more discharge cycles before battery performance begins declining, and greater temperature management requirements and safety^1^. Batteries currently being used in demonstration heavy-duty electric trucks intended for long-distance travel employ technologies that are still in the research and development stage, with uncertain timelines for production scale-up and deployment.

The costs of batteries for electric vehicle applications declined by 16% annually between 2007 and 2019, to an industry-wide average cost of US$161 per kWh for lithium-ion battery packs^16^. For small electric vehicle applications, the Chinese battery manufacturer CATL is developing cobalt-free batteries that are expected to cost US$80/kWh, but it is unclear at what scale these batteries can be produced and what types of applications they will be able to power^17^. There is strong expectation that, with scaled-up production and economies of scale, battery costs will continue to decline significantly over time to the range of US$100/kWh for HDT applications, but there is also uncertainty regarding the forecasted decline because of an expected increase in demand for grid storage and electric vehicles. The cost of batteries will also depend on input raw materials, including lithium, cobalt, nickel sulfate, copper, aluminum, and graphite, whose supply chains could change over time because of increasing demand from other technological applications. A significant increase in demand for batteries to support grid storage for growing renewable power adoption and light-duty electric vehicle deployment is expected, which could impact future prices if there are constraints on raw materials for manufacturing batteries.

Recent improvements are beginning to address the technical barriers related to the limited range and slow charging time of electric vehicle batteries. Heavy-duty electric trucks, such as the Nikola Two and Tesla Semi, have recently demonstrated travel ranges of 400 to 550 miles^3,4^ compared to the 200–300 miles that has been typical until now. There are also early indications that Tesla is working on 30-min fast-charging at a 2C charging rate for its electric Semi truck, with up to 4–6 h of driving time per charge^5^.

Light-duty fuel cell vehicles are commercially available in limited quantities and localized markets in the U.S. and globally, but the technology and markets are still being developed for medium- and heavy-duty vehicle applications. There are only four demonstration Class 8 truck prototypes, manufactured by Hyundai, Toyota, Nikola, and Kenworth. Their ranges extend from a low of 249 miles for the Hyundai XCient Class 8 straight truck (small-scale production began in 2020), to a high of 1000 miles for the Nikola One Class 8 tractor-trailer (production anticipated in 2022)^18^. Scaling up the deployment of hydrogen fuel-cell trucks will depend largely on how this relatively nascent technology matures and when the current high costs associated with the technology and its supporting infrastructure can achieve economies of scale. In addition, technical challenges in hydrogen transportation and distribution networks require harmonized safety and technical standards to support the scale-up of centralized hydrogen production. For hydrogen to be produced from lower-emitting sources, a shift is needed from the current hydrogen production pathways, i.e., from methane reforming of natural gas (76% global share) and coal (23% global share) to electrolysis (2% global share)^1^. Low electricity prices from renewable sources and maximized capacity utilization rates of electrolysers are needed to improve the economic competitiveness of electrolysis based on renewable sources.

Another potential but more uncertain near- to mid-term alternative technology is plug-in hybrid diesel HDTs, which can be a potential lower cost bridge technology for reducing HDT but currently faces market challenges in China. Despite its inclusion in existing NEV subsidy policies, hybrid HDTs only accounted for 0.61% of new NEV HDT sales in 2020 and has not yet gained the attention of domestic HDT manufacturers^19^. In light of its limited CO_2_ emission reduction impact and commercial availability compared to battery electric or fuel cell technologies, we did not consider hybrid HDTs as an alternative in this analysis.

Previous studies have examined the benefits and limitations of NEV technologies for different classes of trucks^3,20–23^ and a growing body of literature is focusing on potential electrification of trucks globally^1,2,24,25^. For China, a limited number of studies have included deployment scenarios of NEV technologies for the entire trucking sector^26–28^ but very few have focused on electrifying HDTs specifically in China^29,30^. The studies that have been done have not considered or compared NEV technologies to other near- and medium-term decarbonization strategies such as energy efficiency, fuel switching, and operations and logistics improvement. We address this research gap by using a technology-rich, bottom-up model to simulate energy consumption and CO_2_ emission impacts of China’s HDTs through 2050. Our analysis examines feasibility, potential applicability, and energy and emissions impacts of multiple decarbonization strategies at different points in time. The results show that introducing battery electric and fuel-cell HDTs as early as 2020 and 2035, respectively, will provide the greatest CO_2_ emissions reduction potential in 2050 by which time the power sector is expected to be significantly decarbonized. However, viable near-term strategies such as improving efficiency, switching to LNG trucks, and improving logistics could halve HDTs’ current diesel consumption and related CO_2_ emissions by 2050.

## Results

### Scenario design

We developed four scenarios: a Reference Scenario that continues existing policies of incremental efficiency improvement and fuel switching; a Short-term Strategies Scenario with full adoption of technical efficiency improvements to engine and vehicle efficiency, commercialized cleaner fuel trucks, and systemic and logistics improvements leading to reduced trucking activity; and two NEV Adoption Scenarios reflecting early and delayed deployment, respectively, of battery electric and fuel-cell HDTs. We used a national bottom-up energy end-use model, China DREAM, based on the Low Emissions Analysis Platform (LEAP) to simulate energy and CO_2_ emissions for HDTs through 2050 for these four scenarios. Sensitivity analysis used additional scenarios to examine the impact of energy intensity changes, activity increases, and earlier adoption of hydrogen fuel-cell instead of electric vehicle technologies. Specific details on each scenario and the sensitivity analysis can be found in the “Methods” section.

### Aggregate heavy-duty truck energy demand and impacts on diesel and natural gas consumption

**Figure 1:**
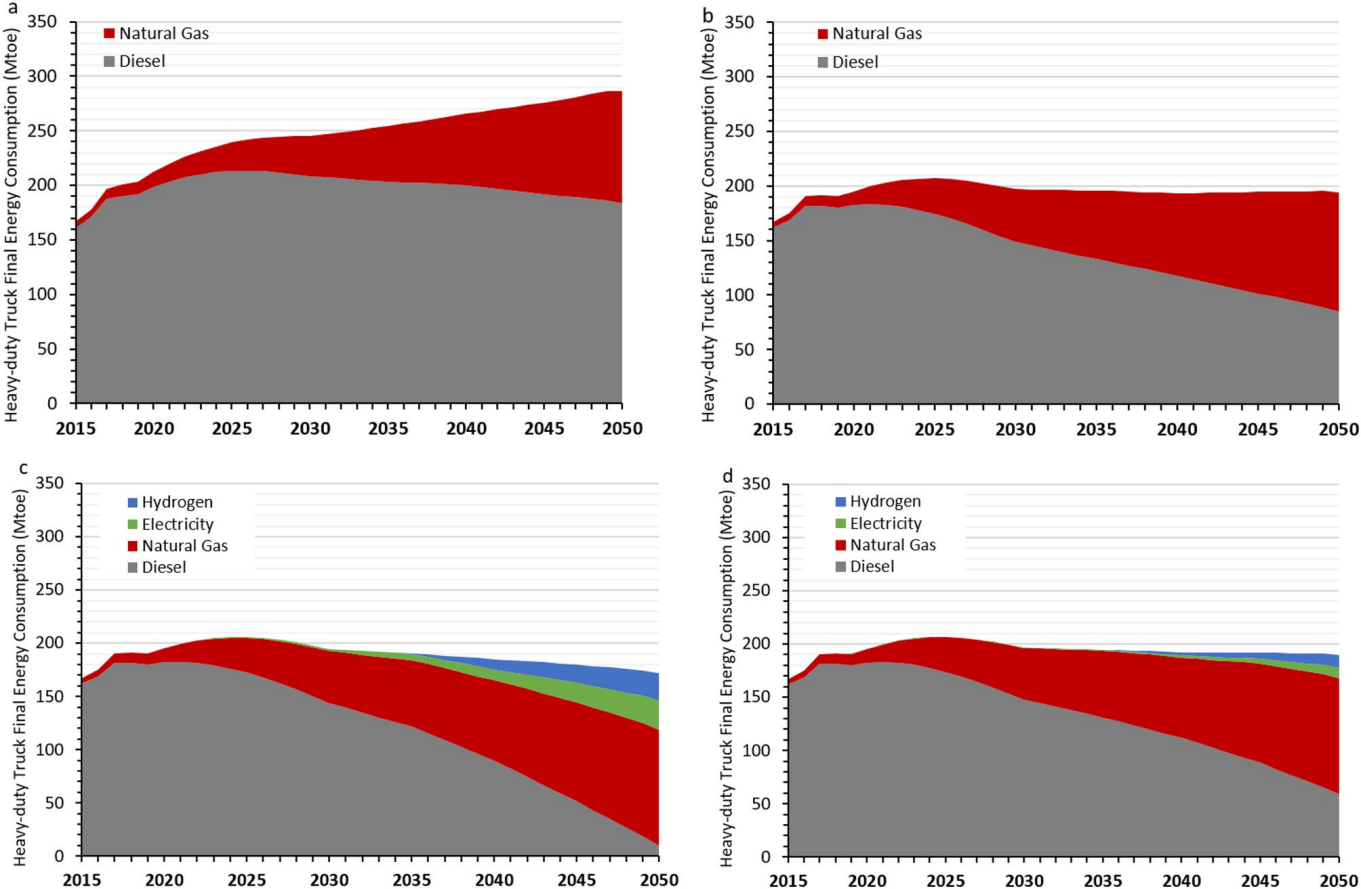
China’s heavy-duty truck fleet final energy consumption results under (**a**) reference scenario; (**b**) short-term strategies scenario with energy-efficiency and logistics improvements and LNG fuel switching; (**c**) NEV early adoption scenario; and (**d**) NEV late adoption scenario.

Based on the latest technological and market trends, we developed four different HDT decarbonization scenarios in China: Reference, Short-Term Strategies, NEV Early Adoption, and NEV Late Adoption. The NEV Early Adoption and NEV Late Adoption scenarios builds on the Short-term Strategies scenario by adding in deployment of NEV HDTs under different timelines. Figure 1 shows the final energy consumption for each.

Under the Reference Scenario, total final energy consumption will continue to grow through the late 2040s. Under the Short-Term Strategies scenario, improvements in efficiency, logistics, and operations will help moderate future growth, with final energy consumption peaking around 2025 and plateauing thereafter despite meeting the same freight transport activity as the Reference Scenario (Fig. 1b). Under the Reference Scenario, diesel consumption will continue to grow through 2026 before declining slowly to a level that is 13% higher than the 2015 level (Fig. 1a). Under the Short-term Strategies Scenario, however, diesel consumption could plateau in the early 2020s and peak by 2025, followed by rapid decline to a 2050 level that is half of total 2015 diesel consumption. This is the combined result of lowered freight activity due to operational improvements and lowered diesel consumption due to efficiency improvements and greater switching towards LNG trucks (Fig. 1b).

Under the NEV Early and Late Adoption Scenarios, total final energy consumption for HDTs will also peak around 2025 and decline (in the case of NEV Early Adoption) or plateau (in the case of NEV Late Adoption) through 2050. Both scenarios will have lower final energy consumption in 2050 compared to the results of the Short-term Strategies Scenario, with smaller increased demand for hydrogen and electricity more than offset by larger reductions in both diesel and natural gas demand (Fig. 1c,d). The overall decline in total final energy consumption under the NEV Early and Late Adoption Scenarios is a result of switching from diesel and LNG trucks with higher energy intensities on the order of 12–15 MJ per vehicle-kilometer travelled, to alternative vehicles with lower energy intensities on the order of 5–10 MJ per vehicle-kilometer travelled. By 2050, diesel demand is nearly eliminated under the NEV Early Adoption Scenario and reduced by two-thirds from 2015 levels under the NEV Late Adoption Scenario. By 2050, hydrogen accounts for 6% and 15% of HDTs’ final energy consumption under the NEV Late and Early Adoption scenarios, respectively, and electricity accounts for 5% and 16% of final energy consumption, respectively.

**Figure 2:**
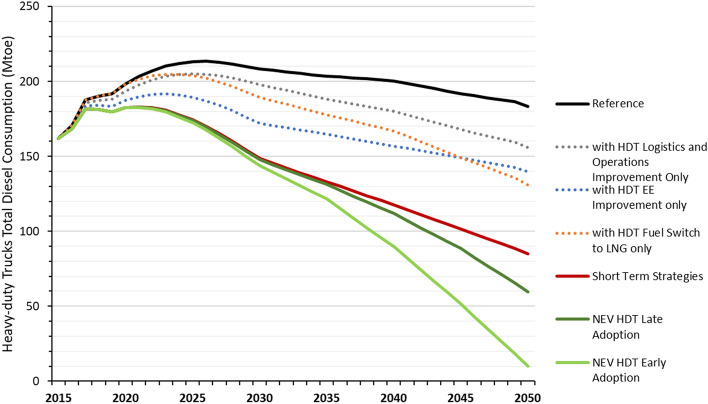
Different strategies’ impact on heavy-duty trucks’ diesel consumption. *EE* energy-efficiency, *NEV* new energy vehicle (battery electric, fuel cell).

Combined, the three short-term strategies of energy-efficiency improvements, fuel switching, and logistics improvements can reduce diesel use by more than half by 2050 from the 2015 level, with consumption peaking and plateauing in the early 2020s (Fig. 2). Individually, energy-efficiency improvements have the largest reduction potential of the three short-term strategies as incremental efficiency improvements on the order of 1.5% per year are possible through advanced engine technologies, improved aerodynamics and lightweight. As a result, efficiency improvements alone can result in diesel consumption peaking in the early 2020s.

Delayed and slower adoption of NEV HDTs, including not introducing fuel-cell trucks until 2040, can still reduce 2050 diesel consumption by an additional 30% (25 million tonnes of oil equivalent or Mtoe) compared to the Short-term Strategies Scenario. This translates into a 45% (88 Mtoe) reduction in 2040 and 68% (124 Mtoe) reduction in 2050 when compared to the Reference Scenario. If NEVs are adopted earlier, with fuel-cell trucks entering the market as early as 2035, then diesel consumption will be further reduced by 55% (110 Mtoe) in 2040 and 94% (173 Mtoe) in 2050 compared to the Reference Scenario. These results show that diesel consumption can be significantly phased out of the heavy-duty trucking sector over time, but that significant reductions beyond existing short-term strategies will not occur until after 2035 when NEV deployment is expected to take off.

**Figure 3:**
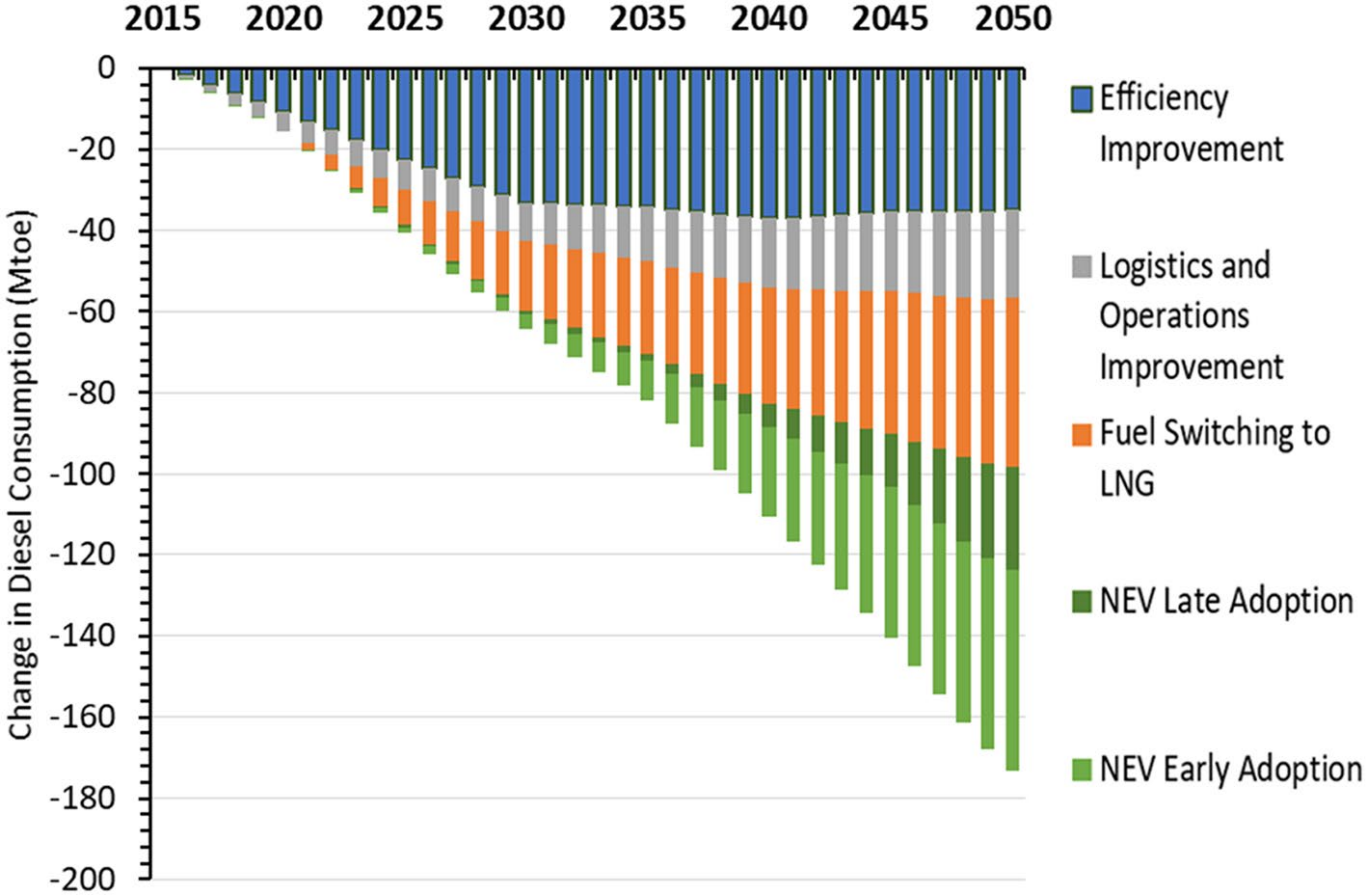
Impact of different decarbonization strategies on heavy-duty trucks’ annual diesel consumption. Change in diesel consumption shown is relative to reference scenario and additive.

Improved efficiency shows the greatest potential to reduce diesel consumption in the near term through the mid-2030s, followed by fuel switching to LNG. In the longer term, from 2030 through 2050, fuel switching to both LNG and clean NEV HDTs will have greater potential to reduce diesel consumption than efficiency improvements alone as more LNG and NEV trucks replace existing diesel trucks (see Fig. 3). The diesel reduction from logistics and operations improvements also increases over time as freight activity grows.

**Figure 4:**
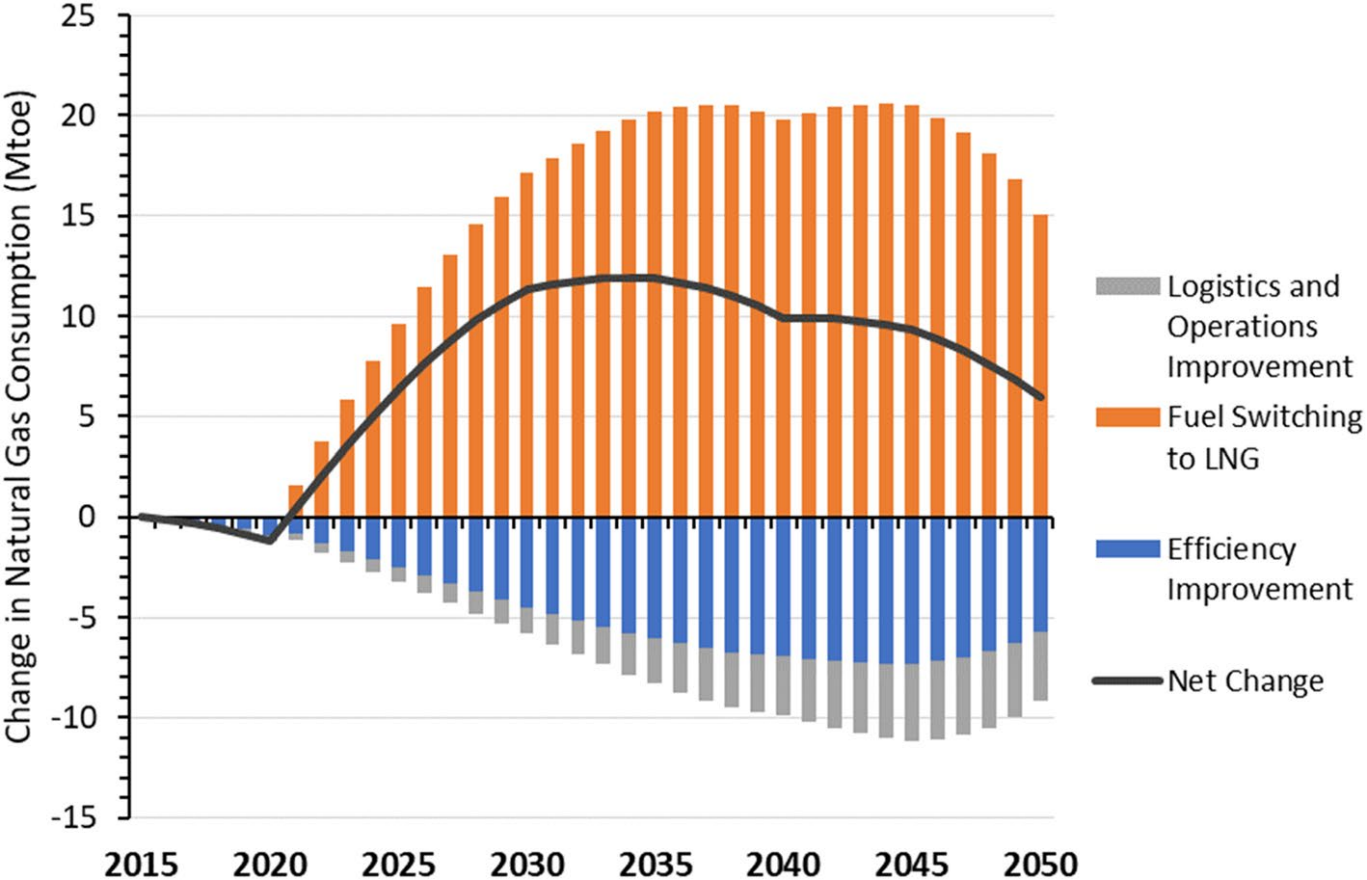
Impact of short-term strategies on heavy-duty truck natural gas consumption.

For natural gas demand, fuel switching to more energy-intensive LNG trucks under the Short-Term Strategies Scenario will increase overall natural gas consumption and offset reductions from improvements in efficiency, operations, and logistics, as seen in Fig. 4. The net increase in natural gas consumption under the combined Short-Term Strategies Scenario climbs to more than 10 Mtoe in the 2030s, the equivalent of nearly one-third of total natural gas consumption under the Reference scenario. The adoption of NEVs does not impact natural gas consumption because only diesel trucks are displaced by NEV trucks.

### Interdependence of new energy vehicles and power sector decarbonization, and overall CO_2_ implications

**Figure 5:**
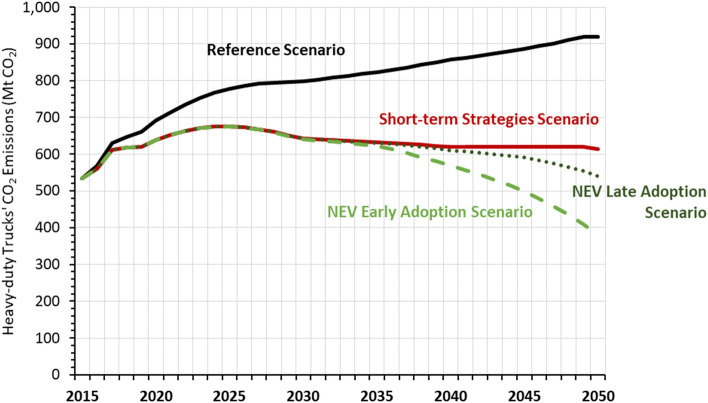
Comparison of heavy-duty truck CO_2_ emissions under reference, short-term strategies, and NEV adoption scenarios.

**Figure 6:**
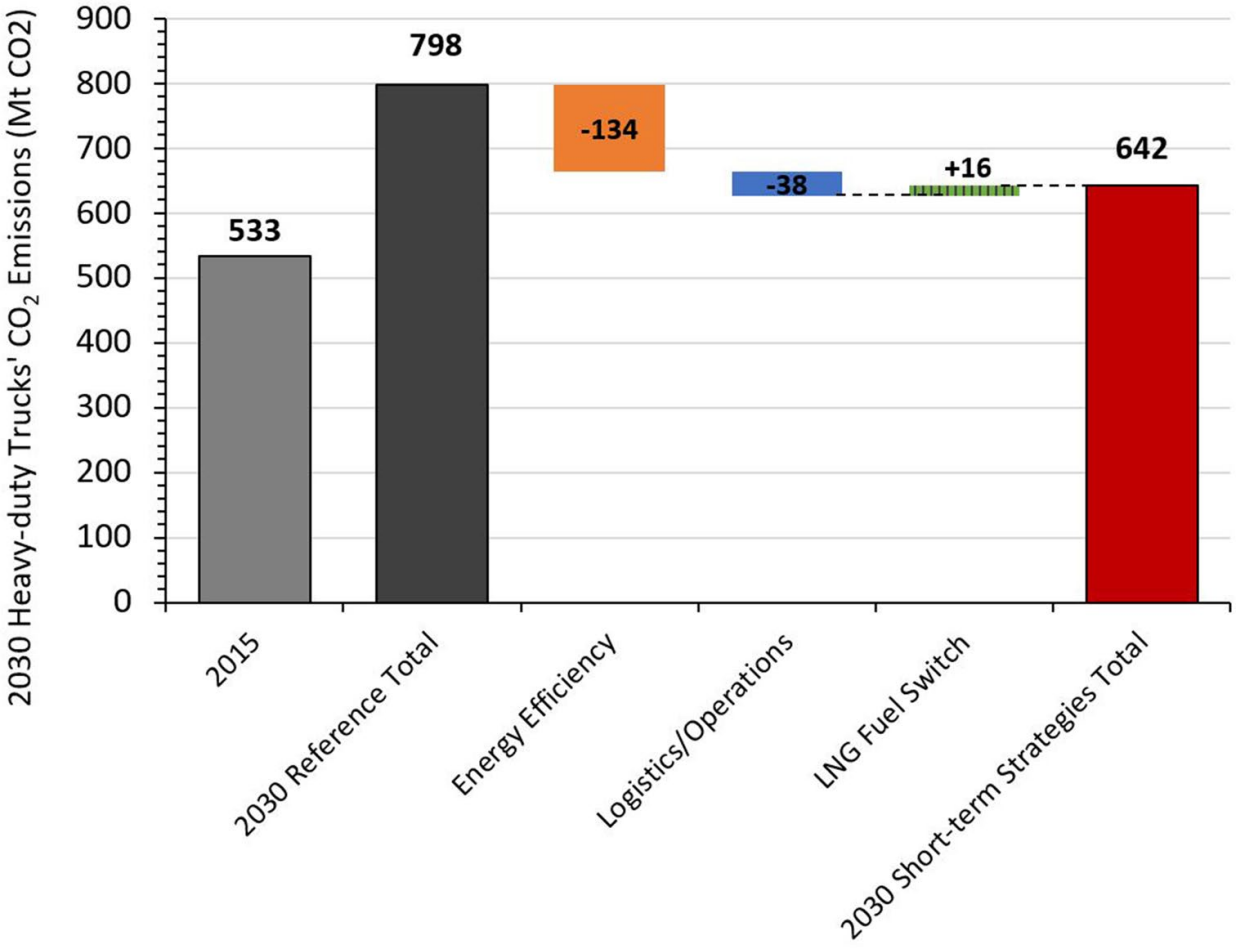
2030 heavy-duty truck CO_2_ emissions impact by short-term decarbonization strategy. Patterned bar denotes net increase in CO_2_ from LNG fuel switch.

HDTs’ CO_2_ emissions peak around 2025 under all three alternative scenarios but not under the Reference scenario (Fig. 5). Despite fuel switching and significant improvements in efficiency, logistics, and operations, CO_2_ emissions from HDTs will remain above 2015 levels in future years unless NEVs are deployed earlier in the NEV Early Adoption Scenario as final energy demand remains higher than 2015 levels. Corresponding to the magnitude of diesel consumption reduction potential, energy efficiency improvement also has the greatest CO_2_ emission reduction potential, followed by logistics and operations improvements in 2030 (Fig. 6). Despite LNG being a cleaner fuel with a lower emissions factor than diesel, switching to LNG trucks will actually result in a small net increase in CO_2_ emissions because LNG’s higher energy intensity offsets reductions in emissions factors, as seen in the increase of 16 MtCO_2_ in Fig. 6 for the year 2030. Additionally, unless there is stringent enforcement of the methane requirements of the China VI emission standards for heavy-duty vehicles, there could be higher than expected tailpipe methane emissions from switching to LNG HDTs^31^.

CO_2_ emissions will peak in 2025 at 675 million tonnes (Mt) of CO_2_ under all three of the alternative scenarios, but the 2050 end-point in CO_2_ emissions varies significantly depending on the pace and scale of NEV adoption (Fig. 5). While CO_2_ emissions plateau under the Short-term Strategies Scenario between 2030 and 2050, CO_2_ emissions continue to decline under both NEV Late and Early Adoption Scenario through 2050 as diesel HDTs are switched to NEVs powered by increasingly cleaner electricity. This assumes an already relatively decarbonized power sector in China where non-fossil sources account for 45% of total electricity generation in 2030, 71% in 2045, and 84% by 2050. As a result of decarbonizing the power sector, the CO_2_ emissions intensity of battery electric trucks falls dramatically from 6.20 kg (kg) CO_2_/kg oil equivalent (kgoe) in 2020 to 2.45 kg CO_2_/kgoe in 2045, compared to diesel emissions intensity of 3.4 kg CO_2_/kgoe and natural gas emissions intensity of 2.8 kg CO_2_/kgoe. Based on NEVs’ significantly lower CO_2_ emissions factor compared to vehicles using other fuels by 2050, early adoption of NEVs can bring significant CO_2_ emissions reductions in 2050, resulting in 30% lower emissions compared to 2015 levels for an HDT fleet that is double the current size.

**Figure 7:**
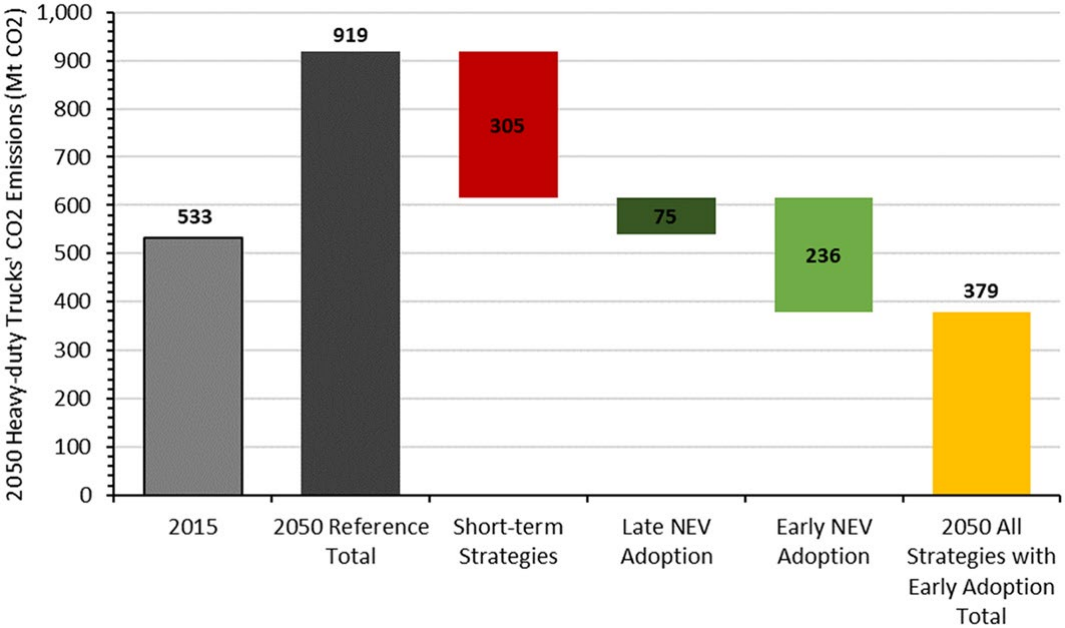
2050 CO_2_ emissions impacts of heavy-duty truck decarbonization strategies.

By 2050, adoption of NEVs will result in notable net CO_2_ emissions reductions for the heavy-duty trucking sector as the power sector significantly decarbonizes (Fig. 7). Adopting NEVs earlier, with subsequently higher shares of battery electric and fuel-cell trucks, will result in significantly greater net CO_2_ emission reductions annually in 2050. Compared to the strategies undertaken in the short term, early adoption of NEV HDTs has the largest (236 MtCO_2_) emissions reduction impact in 2050, followed by energy-efficiency improvements alone with 210 MtCO_2_ emissions reduction potential. In contrast, delaying the adoption of NEV HDTs could have significant impact on future CO_2_ emissions reductions because starting to deploy hydrogen-fuel-cell trucks later, in 2035, will, along with slower battery electric truck adoption, result in much smaller CO_2_ emissions reduction of only 75 MtCO_2_ in 2050.

### Overarching barriers to short and long-term decarbonization strategies

For HDT decarbonization to be realized, China’s automotive industry needs to address significant barriers to the short-term strategies of energy-efficiency improvements, LNG fuel switching, and logistics improvements, as well as to long-term adoption of NEVs. Although the industry has made significant research and development investment and technological improvements in recent years, the fundamental manufacturing processes in China’s heavy-duty vehicle sector still lag behind those of other advanced economies, making it more difficult to adopt high-efficiency and NEV technologies. Key high-end production equipment is lacking, and overall manufacturing quality still needs to be improved, particularly in areas such as high-strength, precision, thin-cast iron casting; carbon fiber structure forming and connection; and precision low-temperature extrusion and forming technologies. Domestic vehicle component manufacturing companies are small in scale with minimal technological capacities, making it difficult for these small manufacturers to produce high-value-added and high-quality components, such as powertrain electronic systems, chassis electronic systems, and super-low friction components. As a result, some key vehicle components are still imported or manufactured by foreign-owned companies in China^27^. China still lags significantly in its adoption of key advanced technologies for HDTs; for example, all U.S. tractor-trailers and 70% of EU tractor-trailers have adopted advanced turbocharging technology, but only 5% of Chinese tractor-trailers have adopted this technology^4^.

China’s trucking industry has distinct institutional characteristics that also influence the potential of short-term decarbonization strategies and NEV truck deployment, including vehicle ownership and decision-making processes, and profit and financing structures. In 2016, a truck driver earned, on average, 100,000 RMB per year, and a new, traditional (internal combustion engine) HDT typically cost about 400,000 to 500,000 RMB. As a result of this profit-to-cost ratio, 84% of truck drivers who were surveyed relied on banks that provide low or zero down payments for truck loans (down payments ranged from 0 to 100,000 RMB) or other informal financing mechanisms^32^.

Moreover, because 71% of the truck drivers own their vehicles, policies or initiatives that increase awareness, provide driver training, and make alternative vehicles more attractive for drivers may be difficult to implement^32^. Being contracted through a logistics company consolidates the trucking industry and can improve operational efficiency, but different logistics companies may have different rules, fees, charges, and requirements. Promoting awareness and aligning goals and objectives across logistics companies therefore becomes another important strategy to increase the potential of NEV HDT deployment.

For both battery electric and fuel-cell technologies specifically, infrastructure development could directly increase or limit the potential for NEV HDT deployment in China. There is concern regarding whether NEV trucks can achieve the 500-mile driving range that is typical of conventional diesel trucks and whether there is variation in actual range due to temperature and grade, load, speed, and installed versus usable battery capacity^33^. As of 2019, China had installed 515,000 public charging stations and 703,000 private charging stations. In 2020, this is estimated to have grown to over 1.7 million total charging stations as a result of new infrastructure stimulus announced in March 2020^34^. Tesla has also installed more than 1000 superchargers for its light-duty vehicles in China, but fewer than 18% of total charging stations in China are fast chargers. HDTs with large battery capacities to support longer driving ranges of 400 miles or more, such as the Tesla Semi, also require a fast charging speed—10 times faster than the current fastest Tesla superchargers. A reasonable charge time of 30 min for HDTs requires a very significant draw from the power grid, with power output greater than 1200 kW^35^. Also, repeated (i.e., 25 times or more) fast charging can damage batteries by causing cracks, leaks, and loss in storage capacity resulting from the battery’s exposure to high temperatures and high resistance^36^. Other key issues related to electric vehicle charging infrastructure development include suboptimal distribution of charging stations, mismatch between demand and supply of electricity for charging, low utilization rates, compatibility issues among charging stations, parking difficulties, and long charging times.

Closely related to the successful deployment of NEV HDTs will be the rapid decarbonization of China’s power sector needed to realize the CO_2_ emission reduction potential of increased NEV HDT adoption. We assumed non-fossil generation will increase to account for 45% share by 2030, up from reported 32% share in 2020^37^, and rise to 84% by 2050, but deep decarbonization of the power sector will require significant policy support and continued power sector reform. Although China is the world’s leader in renewable capacity growth, China still has the world’s largest coal-fired fleet of over 1000 GW with long remaining lifetimes for most coal plants^38^. Government-driven long-term vision and continuous policy support have helped transform China’s power mix in a short-period of time, but wide-ranging market reforms and coal phase-out are still needed to achieve rapid deep decarbonization^39^. Possible strategies for phasing-out coal power could include cancelling planned projects, shutting down a subset of existing but poor performing plants, and reducing hours for remaining plants to mainly meet peak load demand^38^. At the same time, modernizing grid transmission and distribution and developing storage and reducing institutional barriers to inter-regional power trade can help improve renewable integration while addressing intermittency challenges^40^.

Our scenarios assume that the barriers identified above will be addressed through policies, programs, and market-based changes, but the timing of removal of these barriers will determine the cumulative diesel and CO_2_ reductions from decarbonizing HDTs.

## Conclusions

Our results suggest that under a Short-Term Strategies Scenario, diesel consumption could plateau in the early 2020s and peak by 2025, followed by rapid decline to a 2050 level that is half of 2015 total diesel consumption. Of the strategies considered for the short term, energy-efficiency improvement has the greatest potential for significantly reducing diesel consumption and can, by itself, enable diesel consumption to peak in the early 2020s. In the longer term, fuel switching to both LNG and NEV HDTs will have greater, and approximately equal, potential for reducing diesel use as LNG and NEV truck sales ramp up, in combination with savings from systemic improvements in logistics and operations. The delayed, slower adoption of NEVs for HDTs, including not introducing fuel-cell trucks until 2040, further reduces 2050 diesel consumption by 30% when compared to the Short-Term Strategies Scenario. Earlier adoption of NEVs before 2035 can reduce 2050 diesel consumption by 94% when compared to the Reference Scenario. Natural gas consumption increases under the Short-Term Strategies Scenario because of fuel-switching to higher-energy-intensity LNG trucks, resulting in additional demand of more than 1 Mtoe in the 2030s.

From the perspective of CO_2_ emissions, short-term strategies have the most significant impact on overall CO_2_ reductions in the earlier years, with greatest impact resulting from efficiency improvements, followed by logistics improvements and LNG fuel switching (which has a lower CO_2_ emissions factor that is offset by higher energy intensity per kilometer traveled). Together, these strategies can help contribute to meeting China’s newly announced goal of peaking its CO_2_ emissions before 2030. However, short-term strategies will result in a net increase in natural gas consumption, despite overall efficiency and logistics improvements, which could exacerbate China’s current natural gas import dependency concerns even though total diesel consumption is reduced. Ramping up the adoption of either electric or fuel-cell trucks from 2030 to 2050 will have similar impacts on total final energy consumption and CO_2_ emissions, but existing technological, cost, and infrastructure development barriers are greater for scaling up fuel-cell truck deployment. However, earlier adoption of NEVs will result in much larger net CO_2_ emissions reductions over time, if the power sector continues to be significantly decarbonized. By 2050, annual CO_2_ emissions reductions from the early adoption of NEV HDTs (i.e., battery electric trucks beginning in 2020, hydrogen fuel-cell trucks beginning in 2030s) will be more significant, on the order of 235 Mt CO_2_, and have the greatest reduction potential, followed by energy-efficiency improvements as a viable short-term strategy. Delaying the adoption of NEV HDTs could have significant impact on future CO_2_ emissions reductions because starting to deploy hydrogen-fuel-cell trucks later, in 2035, will, along with slower battery electric truck adoption, result in much smaller CO_2_ emissions reductions even as late as 2050.

These findings underscore the importance of developing policies and programs that support a diverse mix of technical and technological solutions to mitigate the CO_2_ and broader environmental impacts of an HDT fleet that is currently dominated by diesel technologies. To achieve the maximum CO_2_ and diesel reductions and support China’s efforts to achieve carbon neutrality by 2060, decarbonization of HDTs needs to rely on a mix of near- and longer-term strategies and technologies rather than one single NEV technology. Although existing technical improvements in energy efficiency and adoption of readily available cleaner LNG trucks can lead to sizable reductions in diesel consumption in the near term, additional policy support is needed to accelerate research and development on NEV technologies and build the foundation to expand NEV charging infrastructure for the longer term. This can be achieved by adopting technology-neutral performance standards for greenhouse gas reduction instead of mandates that pick a technology, or fiscal policies such as reduced excise taxes and import tariffs on zero-emissions HDTs to help promote the market entry and adoption of newly launched NEV technologies in China. A dual policy focus on both near-term action and longer-term planning to enable ramp-up in adoption of NEVs over the next decade is needed because CO_2_ emissions reductions will be difficult to sustain until 2050 if adoption of NEVs is delayed past 2035.

## Methods

### Modelling methodology

The analysis of heavy-duty road freight decarbonization strategies uses the Berkeley Lab’s China 2050 Demand Resource Energy Analysis Model (DREAM), a national bottom-up energy end-use model that includes energy demand, supply, and transformation sectors. This model is an application of the Low Emissions Analysis Platform (LEAP) developed by the Stockholm Environmental Institute^41^. Previous applications of this model on different sectoral analyses and national energy and emission pathways were evaluated by others^42,43^.

Key macroeconomic parameters that drive energy-using activity such as economic growth, population, and urbanization are aligned with international sources as well as Chinese sources^44,45^. The average annual GDP growth rate used in this analysis is 6.5% (2015–2030) and 3.4% (2030–2050), while population increases 6% annually between 2010 and 2020 and declines 4% annually between 2020 and 2050.

Transport sector activity is driven by demand for freight transport and for passenger transport. Equation (1) summarizes the variables in the calculation for transport energy consumption in China 2050 DREAM.

1 $$ E_{TR} = \sum\limits_{k}^{OPTION} {\sum\limits_{t}^{OPTION} {\sum\limits_{r}^{OPTION} {\sum\limits_{j}^{OPTION} {Q_{t,r,m,i} \times f_{k,t,r,j,i} \times EI_{TR,k,t,r,j,i} ,} } } } $$

where *j* = transport technology class (e.g., vehicle classes), *f*_*k,t,m,j*_ = share of fuel *k* used for technology *j* in providing transport services of type *t*, *r* = mode type (road, rail, water, air, pipeline), *m* = locale type (intra-city, inter-city, international water), *Q*_*t,r,m*_ = quantity of transport service of type *t* in mode *r* and in locale *m* of region *i* in veh-km, and *EI*_*TR,k,t,,m*_ = average energy intensity of energy type *k* for transport service of type *t* in mode *r* and in locale *m* in MJ/(veh-km-year), *k* = fuel type, *t* = transport type (passenger, freight).

For heavy-duty trucks, we use a bottom-up stock turnover model to project future sales and the implied stock of heavy-duty trucks. We calibrate historical sales and stock using the China Statistical Yearbook through 2018, and project future sales and stock assuming an average lifetime of 10 years through 2050^46^. Where data was available, we took into consideration different energy-related characteristics for different vehicle sub-categories such as tractor trailers, special trucks, dump and trucks, including specific fuel shares and specific fuel consumption rates for each sub-category^7^. However, due to data limitations, we did not attempt to model different operating characteristics of each sub-category of heavy-duty trucks such as annual distance traveled or other operating characteristics. Instead, we used data for tractor trailer trucks—the second most common type of heavy-duty trucks and most likely to grow in the future—as a representative type for all heavy-duty trucks. Total freight activity in terms of vehicle-kilometers travelled are projected based on the assumptions and sources listed in Table 1.

**Table 1:** Key assumptions for heavy-duty truck activity and energy projections.

	2015–2050 assumptions	Sources
Vehicle average lifetime	10 years	^2,47^
Annual average vehicle kilometers travelled	Intra-city: 50,000 km/vehicle/year	^8^
	Inter-city: 100,000 km/vehicle/year	
Inter- and intra-city travel shares	Intra-city: 10%	Based on HDT driving cycle standard^48^
	Inter-city: 90%	
Total stock	2015: 5.3 million	Calculated by bottom-up stock turnover model
	2050: 10.5 million	
Fuel shares	2015: 97.6% diesel, 2.4% liquefied natural gas	Weighted-average shares of all heavy-duty truck types from Ref.^7^
	Projected fuel shares vary by scenario (see below)	
Final energy intensity	2015 diesel trucks: 38.3 L/100 km (13.8 MJ/km)	Weighted-average fuel consumption of all heavy-duty truck types as reported in Ref.^7^
	2015 LNG trucks: 81.3 L/100 km (18.3 MJ/km)	
	Projected intensities vary by scenario as discussed below	

On the supply side, the energy transformation sector includes a power-sector module that can be adapted to reflect changes in generation-dispatch algorithms, efficiency levels, generation mix, and demand-side management. Table 2 shows the assumed installed capacity by different power generation technologies used to model the power sector for all four scenarios.

**Table 2:** Key installed capacity assumptions for power sector.

Unit: GW unless noted otherwise	2015	2020	2025	2030	2035	2040	2045	2050
Coal	908	855	911	904	894	833	654	442
Natural gas	66	110	136	161	194	225	258	294
Diesel	4	11	11	11	11	10	8	6
Nuclear	27	58	83	100	131	155	192	221
Biomass	11	20	25	30	31	32	33	34
Hydro	320	380	395	410	425	444	474	501
Solar	42	269	299	383	675	1023	1451	1920
Wind	131	260	397	482	774	1002	1142	1217
Geothermal	0	0	0	0	0	0	1	1
Total installed capacity	1510	1963	2257	2482	3135	3725	4213	4637
Non-fossil % of installed capacity	33%	47%	49%	53%	61%	67%	74%	79%
Non-fossil % of electricity generation	24%	42%	43%	45%	53%	60–62%*	71–74%*	84–90%*

*Non-fossil % of electricity generation varies by HDT scenario described below.

The power generation sector models different power generation technologies including coal, natural gas, biomass, nuclear, wind, hydro, solar, and geothermal power generation. Following specified power sector module parameters, the model uses algorithms to calculate the amount and type of capacity required to be dispatched to meet the final electricity demand from the economic sectors. For this study, the model follows an environmental or green dispatch order that dispatches generation based on their environmental (i.e. low carbon) merit by prioritizing non-fossil generation before fossil generation for dispatch to meet electricity demand from the demand sectors. Energy-related CO_2_ emissions are calculated by the model using China-specific energy content for fossil fuels and IPCC emission factors.

Due to time lags in national energy-related data reporting at the sectoral level, the model’s base year is set at 2015. However, all model inputs with historically reported time series such as heavy-duty truck sales and stock have been updated through the latest available year (2018 in most cases) and the transport sector’s total energy balance has been calibrated with model outputs up to 2018.

## Data and scenarios

This study uses four main scenarios to evaluate the potential pathways for China’s heavy-duty freight sector to reduce oil consumption through continuous improvement at recent pace of improvement, deployment of short-term strategies for accelerated efficiency, fuel switching and logistics improvement, and two scenarios of accelerated adoption of alternative clean energy vehicle technologies. The overall activity drivers and total activity in terms of projected vehicle stock and distance traveled is the same for all three scenarios, but each scenario differs in terms of the projected technology mix and levels of efficiency.

These specific scenarios include:

1. *Reference scenario* of continuous efficiency improvement (0.6–0.7%/year) and fuel switching to LNG (30% by 2050) consistent with the pace of policy-driven change in recent years. In the absence of additional policies pushing for freight electrification, battery electric or hydrogen fuel cell trucks are not expected to penetrate the heavy-duty trucking sector through 2050.
2. *Short-term strategies scenario* as an alternative pathway in which China adopts all technical efficiency improvements, cleaner fuel trucks that are commercialized and readily available, and systemic improvements in heavy-duty freight operations and logistics at an accelerated pace to reduce the total energy, and particularly oil consumption of the heavy-duty freight sector. In the model, this translates into greater energy efficiency improvements (1.4–1.5%/year), higher adoption of commercialized cleaner fuel trucks (50% LNG vehicles by 2050), and systemic and logistics improvements leading to 15% reduction in heavy-duty freight activity by 2050.

Building on the short-term strategies scenario and considering ongoing challenges and the relatively high level of uncertainty surrounding both the pace of technological advancement and policy and program development to resolve existing barriers to market adoption of NEV trucks, two additional scenarios are used to represent two potential pathways for accelerating the deployment and adoption of battery electricity and hydrogen fuel cell truck.

3. *Early NEV adoption* with earlier and accelerated deployment of battery electric and fuel cell heavy-duty trucks starting in 2020 and 2035, respectively, reaching 30% and 10% of the entire heavy-duty fleet in 2050, respectively. The non-linear growth trajectory in the market adoption of fuel cell heavy-duty trucks is consistent with existing projections of hydrogen fuel-cell technology penetration in China’s total road vehicle fleet under optimistic scenarios from Liu et al. For battery electric trucks, the market adoption rate is assumed to be roughly twice as fast as fuel cell trucks based on the current faster pace of technology development, including specifically the many more existing models of demonstration electric heavy-duty truck models and pilot projects globally as well as shorter anticipated timeline for commercial production by major manufacturers and existing charging infrastructure roll-out plans.
4. *Late NEV adoption* of battery electric and fuel cell heavy-duty trucks that start later in 2020 and 2040, respectively, and at a slower pace through 2050 to reach 14% and 5% of the truck fleet, respectively. The non-linear growth trajectory in fuel cell heavy-duty truck adoption is consistent with existing projections of hydrogen fuel-cell penetration in China’s total road vehicle fleet under more conservative scenarios^49^.

In addition to rising market shares, the average energy efficiency of both battery electric and fuel cell heavy-duty trucks is assumed to improve over time as a result of technological improvement and at the same rate under both the Early and Late Adoption Scenarios. For hydrogen fuel cell heavy-duty trucks, the stock average energy intensity declines from 2015 levels of 10 kg H2/100 km based on existing prototypes to 7.5 kg H2/100 km based on existing projections of hydrogen consumption rates for commercial vehicles in China^27^. Because it is a stock-average value, the 2050 intensity is slightly higher than today’s best available intensity values of 6 kg H_2_ per 100 km in current prototype trucks. For battery electric heavy-duty trucks, the stock-average improves from the current average value of 1.5 kWh per kilometer^35^ to today’s best available intensity of 1.25 kWh per kilometer announced for the Tesla Semi tractor trailer by 2050.

For hydrogen production for fuel cell battery trucks, future hydrogen production in China is assume to remain split 50%/50% between methane reforming from natural gas and electrolysis through 2050. Electrolysis is directly linked to the fuel mix of the entire power sector, which becomes increasingly decarbonized over time, whereas methane reforming remains dependent on natural gas as the feedstock fuel. Because large-scale economic hydrogen production assumes high capacity factors for electrolyzers, it would not be economic to rely only on periodic surpluses in renewable electricity for batch production.

## Sensitivity analysis

In light of the uncertainty surrounding some key future drivers affecting energy demand and energy-related CO_2_ emissions, particularly for future heavy-duty truck activity, pace of energy efficiency improvement, and adoption of NEVs, sensitivity analysis was conducted to measure the impact of key parameters as shown in Table 3.

**Table 3:** List of sensitivity scenarios.

Scenario	Input parameter sensitivity
Frozen energy intensity	No change in HDT energy intensities to set maximum upper bound for energy consumption given existing activity growth and fuel share projections
Lower energy intensity reduction	Tests 50% of modeled energy efficiency improvement under Short-term Strategies Scenario, to account for uncertainties in future technological or policy progress
Higher fleet activity	Tests 50% increase in total fleet activity (i.e., vehicle-kilometers travelled) to account for uncertainties in faster rise in vehicle stock, higher than projected average distance travelled per vehicle, or a combination of both
NEV HDT early adoption of hydrogen fuel cell	Tests the earlier and accelerated adoption of hydrogen fuel cell heavy-duty trucks instead of accelerated adoption of electric heavy-duty trucks due to uncertainties in technological adoption. Assumes that hydrogen fuel cell trucks will reach 30% of the heavy-duty stock by 2050, with electric trucks only reaching 15% by 2050
NEV HDT early adoption of plug-in hybrid	Tests the alternative earlier adoption of plug-in hybrid diesel heavy-duty trucks instead of battery electric trucks due to potential cost and range advantages. Assumes that plug-in hybrid HDT adoption will follow same adoption rates as battery electric through 2040, and reach 20% share of heavy-duty stock by 2050

**Figure 8:**
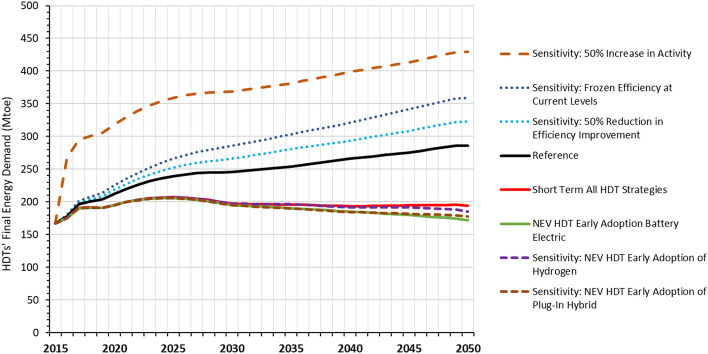
China’s HDTs’ final energy demand results by sensitivity analysis scenario.

The sensitivity analysis finds that changing the activity level by 50% has a very significant impact on the final energy demand of heavy-duty trucks, leading to 2.5 times greater energy demand in 2050 compared to the current levels (Fig. 8). In contrast, freezing energy efficiency improvements will only increase final energy demand in 2050 by 26%, compared to the 2050 Reference level, while 50% reduction in efficiency improvement will result in an increase of only 13% in 2050 final energy demand. This suggests that uncertainties over projected increase in truck fleet and/or distance traveled could have greater impact on oil demand than uncertainties in the degree of technological progress in efficiency improvements. For early adoption of NEVs, whether the accelerated adoption is of hydrogen fuel cell, battery electric or plug-in hybrid electric heavy-duty trucks has very little impact on final energy demand. Earlier adoption of hydrogen fuel cell trucks or plug-in hybrid trucks result in slightly high final energy demand than earlier adoption of battery electric trucks, but the total energy demand remains slightly lower than the Short-term Strategies scenario without NEVs. While plug-in hybrid electric HDT adoption can contribute additional CO_2_ emission reductions beyond the Short-term Strategies scenario, its magnitude of CO_2_ emissions reductions will be lower than battery electric and hydrogen fuel cell HDT adoption.

## Data Availability

The data that supports the plots within these paper and other findings of this study are available from the corresponding author upon reasonable request.
